# Loss of Imprinting of *Cdkn1c* Protects against Age and Diet-Induced Obesity

**DOI:** 10.3390/ijms19092734

**Published:** 2018-09-12

**Authors:** Mathew Van de Pette, Simon J. Tunster, Rosalind M. John

**Affiliations:** School of BioSciences, Cardiff University, Cardiff CF10 3AX, UK; matthew.van-de-pette@imperial.ac.uk (M.V.d.P.); sjt95@cam.ac.uk (S.J.T.)

**Keywords:** *Cdkn1c*, imprinting, BACs, adipose, aging, high fat diet, methylation

## Abstract

*Cyclin dependent kinase inhibitor 1c* (*Cdkn1c*) is a maternally expressed imprinted gene with roles in embryonic development, post-natal metabolism and behaviour. Using mouse models with altered dosages of *Cdkn1c*, we have previously identified a role for the gene in promoting brown adipose tissue formation. Here, we use these transgenic mouse lines to model the loss of imprinting of *Cdkn1c* in adulthood. We demonstrate that only a two-fold increase in the expression of *Cdkn1c* during development is sufficient to protect against age-related weight gain in addition to glucose and insulin intolerance. Further to this, we show that the loss of imprinting of *Cdkn1c* protects against diet-induced obesity. Bisulphite sequencing was performed to test the stability of the two differentially methylated regions that regulate *Cdkn1c* imprinting, and both were found to be unaltered in aged or diet-challenged adipose tissue, despite drastic reductions in *Cdkn1c* expression. These data demonstrate a critical role for *Cdkn1c* in regulating adult adipose tissue, with modest changes in expression capable of protecting against both age and diet-induced obesity and metabolic syndrome, with a natural decline in *Cdkn1c* expression observed that may contribute to less healthy metabolic aging. Finally, we have observed a post-natal insensitivity of the imprint to environmental factors, in contrast to recent observations of an *in utero* sensitivity.

## 1. Introduction

Genomic imprinting is a phenomenon of epigenetic gene regulation, whereby mono-allelic expression of a gene occurs, in a parent-of-origin specific manner in placental mammals and some plants [1,2]. *Cyclin dependent kinase inhibitor 1c* (*Cdkn1c*) is a maternally expressed imprinted gene found on the IC2 imprinting cluster on mouse chromosome 7 [3]. The gene functions both in limiting cell proliferation, primarily as a G1/S phase inhibitor [4,5], and in directing differentiation of certain select lineages. These include regulating the differentiation of skeletal muscle [6], amacrine cells in the retina [7], and the maintenance of adult quiescent neural stem cell populations [8,9]. Imprinted expression of *Cdkn1c* is achieved through two distinct differentially methylated regions (DMRs). A germline differentially methylated region termed *KvDMR* is located >200 kb upstream of *Cdkn1c* within the *Kcnq1* gene, and this region acquires DNA methylation in the maternal germline in mice and humans to establish imprinted expression of an extensive genomic region on mouse distal chromosome 7/human chromosome 11p15, called the IC2 cluster [10,11,12]. The *Cdkn1c somatic DMR* (*sDMR*) is located at the 5′ terminus of the *Cdkn1c* coding region, and is required for silencing maintenance, as opposed to establishment, through methylation of the silent paternally inherited allele [13]. This region has been shown to only possess methylation in somatic tissue in vivo, acquired during early embryonic development and after allelic expression is established [14,15,16]. Our recent work using an imaging-based model of *Cdkn1c* expression reported that the *Cdkn1c sDMR* is sensitive to gestational protein restriction, with offspring exposed to the diet found to have reduced embryonic and post-natal methylation at this region, with an accompanying loss of imprinting of *Cdkn1c* [17].

Elevated expression of *Cdkn1c* has been implicated as a cause of two similar but distinct childhood growth restriction disorders, Silver–Russell (SRS) and IMAGe syndrome [18], while a loss-of-function of *Cdkn1c* is present in familial cases of the childhood overgrowth disorder Beckwith–Wiedemann syndrome [19]. In order to model SRS, our lab developed transgenic mouse lines carrying additional copies of *Cdkn1c*, on a bacterial artificial chromosome (BAC) spanning *Cdkn1c* and two other imprinted genes *Phlda2* and *Slc22a18*. To distinguish between the effects of elevated *Cdkn1c* expression and the effect of increasing the dosage of the other two genes, we developed a transgenic line containing a modified version of this same BAC where *Cdkn1c* expression is disrupted by a *β-galactosidase* insertion [20,21]. These lines provide an alternative route for studying imprinted genes, focusing on dosage rather than gene function *per se*. Classical knockout models, used to study the gene function of imprinted genes, frequently present with high levels of lethality associated with heterozygous deletion models presenting as a phenotypic homozygous deletion, due to the mono-allelic expression [22]. Using the BAC transgenic lines, we have demonstrated a dosage-dependent fetal growth restriction due to elevated *Cdkn1c* [20], which was maintained post-natally and into early adulthood. Further to this, a new function of the gene in regulating brown adipose tissue formation was recently described by our group, with elevated markers of mitochondrial uncoupling also uncovered in the white adipose tissue [23]. In SRS, children are born small and fail to catch up, with excessive thinness being an additional characteristic. Some SRS children are also reported to be fussy eaters, which has been suggested to contribute to their failure to gain weight. However, we showed that young mice carrying an extra copy of *Cdkn1c* were also thin with little subcutaneous fat despite consuming similar calories to controls [23]. Some SRS children also have night sweats which could suggest dysfunctional thermoregulation, consistent with the increase in brown adipose tissue we observed in our mouse model [23].

In this study, we further explored the impact of increased *Cdkn1c* dosage in adult mice, focusing on the predicted role for *Cdkn1c* in influencing adult weight gain through regulating the development of brown fat depots. Imprinted gene function has classically been thought to be predominantly restricted to regulating embryonic and placental development; however, recent work has highlighted important post-natal functions for this class of gene [23,24,25]. Therefore, further elucidation of both gene and imprint function, in adulthood, will enable a clearer understanding of this method of epigenetic gene regulation.

## 2. Results

**Figure 1:**
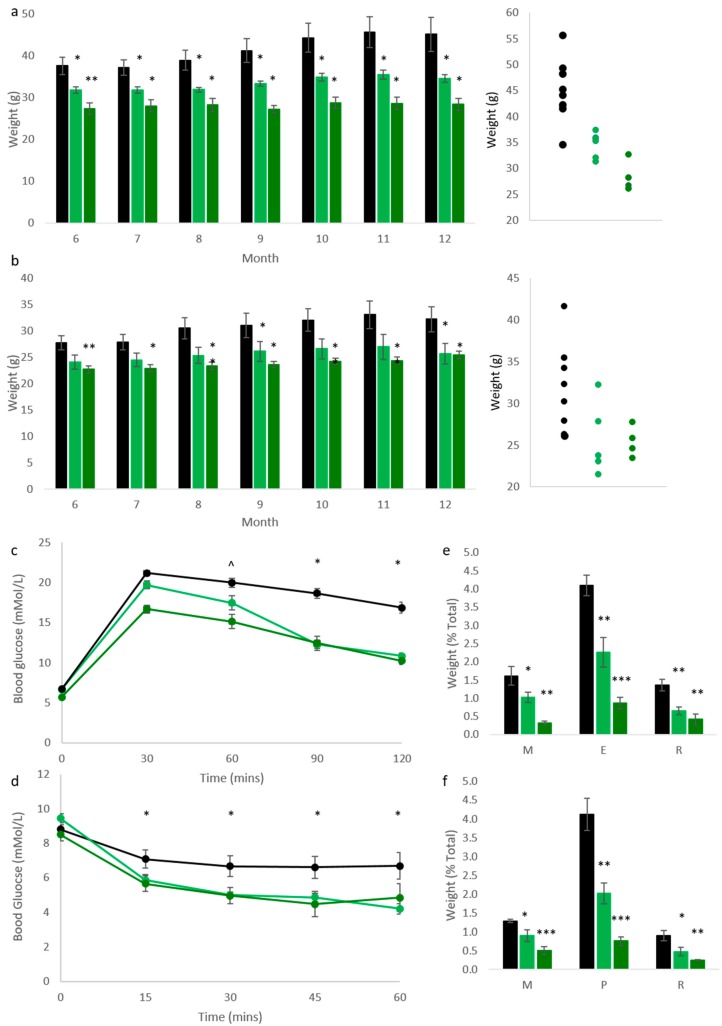
Increased *Cdkn1c* dosage protects against age-related metabolic disorder. (**a**) Adult whole-body weights. Both Cdkn1c^BACx1^ (single copy bacterial artificial chromosome (BAC), light green) and Cdkn1c^BACx2^ (double copy, dark green) transgenic males were significantly lighter than wild type (black) litter mates between 6 months and 1 year of age, in a dosage-dependent manner. Individual 12-month weights are displayed on the right-hand graph (n: WT 8, Cdkn1c^BACx1^ 6, Cdkn1c^BACx2^ 4), * *p* < 0.05, ** *p* < 0.01; (**b**) Cdkn1c^BACx1^ (light green) and Cdkn1c^BACx2^ (dark green) transgenic females were also found to be significantly lighter than wild type (black) litter mates between 6 months and 1 year of age. Individual 12-month weights are displayed on the right-hand graph (n: 9, 5, 4), * *p* < 0.05, ** *p* < 0.01; (**c**) Glucose tolerance testing of 1-year old males revealed an improved clearance of glucose challenge in both Cdkn1c^BACx1^ and Cdkn1c^BACx2^ lines when compared to wild type mice. (n: 8, 6, 4), * *p* < 0.05, ^ *p* < 0.05 (Cdkn1c^BACx2^ only); (**d**) Insulin tolerance testing of 1-year old males revealed greater sensitivity to insulin in both Cdkn1c^BACx1^ and Cdkn1c^BACx2^ lines when compared to wild type mice, (n: 8, 6, 4), * *p* < 0.05; (**e**) Wet weight of white adipose tissue in 1-year old males, as a percentage of total body weight. Mesenteric (M), epididymal (E) and retroperitoneal (R) depots were all found to be significantly smaller in Cdkn1c^BACx1^ and Cdkn1c^BACx2^ males compared to wild type. (n: 8, 6, 4), * *p* < 0.05, ** *p* < 0.01, *** *p* < 0.001; (**f**) Wet weight of white adipose tissue in 1-year old females, as a percentage of total body weight. Mesenteric (M), peri-ovarian (P) and retroperitoneal (R) depots were all found to be significantly smaller in Cdkn1c^BACx1^ and Cdkn1c^BACx2^ males compared to wild type, in a dosage-dependent manner. (n: 9, 5, 4). * *p* < 0.05, ** *p* < 0.01, *** *p* < 0.001.

We have previously reported that mice bearing one (Cdkn1c^BACx1^) or two copies (Cdkn1c^BACx2^) of a BAC spanning the *Cdkn1c* locus were significantly lighter than wild type (WT) litter mates at 10 weeks of age, with relative increases in expression of *Cdkn1c* in white and brown adipose tissue [23]. This phenotype was attributed to excess *Cdkn1c*, causing jointly a fetal growth restriction phenotype that was not resolved post-natally and reduced white adipose tissue (WAT) stores due to elevated brown adipose tissue (BAT) formation. To further understand the effect of elevated *Cdkn1c* into adulthood, mice were aged to 1 year, co-housed with sex-matched WT littermates. Monthly weighing found significantly reduced weights in both male (Figure 1a) and female (Figure 1b) Cdkn1c^BACx1^ (light green) and Cdkn1c^BACx2^ (dark green) mice when compared to wild type (black). In fact, all four transgenic groups increased in weight by less than 3 g over this 6-month period, in contrast to an average 7.5 g increase in male, and 4.5 g increase in female WT. Glucose and insulin tolerance testing was performed on the male cohort at 1 year of age, and found a significantly improved clearance of glucose after challenge (Figure 1c) in both Cdkn1c^BACx1^ and Cdkn1c^BACx2^ lines, in addition to heightened response to insulin testing, compared to age-matched WT males (Figure 1d). White adipose deposits were weighed in culled mice and found to be significantly smaller in both males (Figure 1e) and females (Figure 1f), as a percentage of total body weight, in order to control for growth restriction. As had been observed in the whole-body weights (Figure 1a,b), this reduction was found to be dosage-dependent, with Cdkn1c^BACx2^ mice showing a greater reduction in fat pad weights when compared to Cdkn1c^BACx1^.

**Figure 2:**
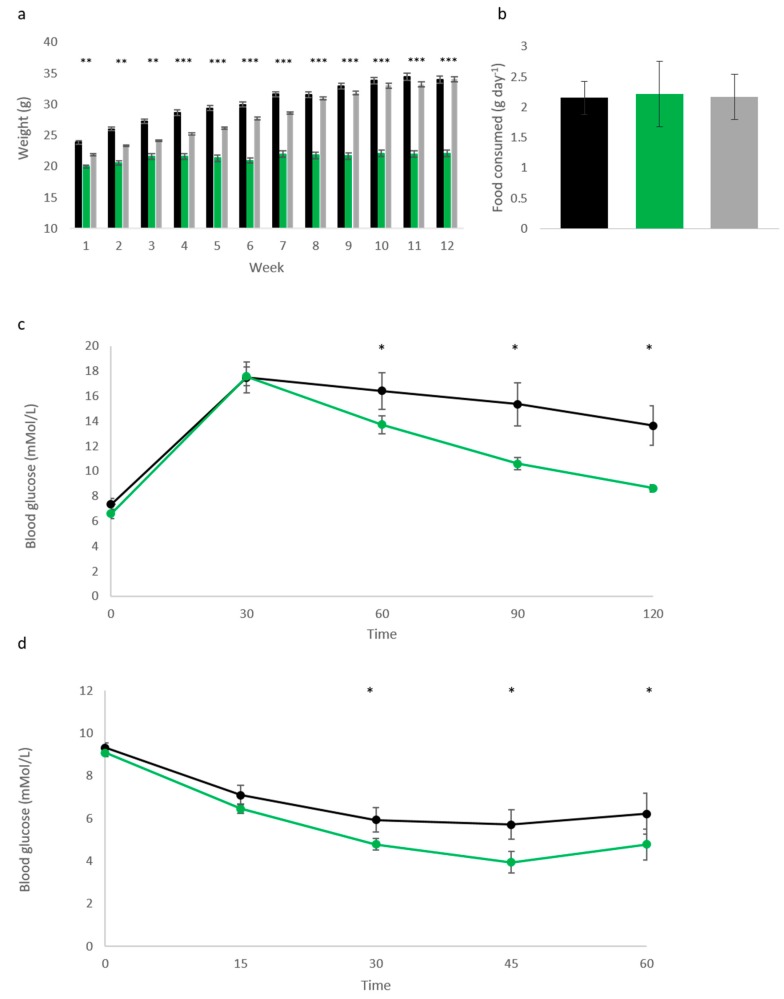
Increased *Cdkn1c* dosage protects against diet-induced obesity. (**a**) Cdkn1c^BACx1^ male mice (light green) were found to resist weight gain during a 12-week high fat diet challenge, while wild type (WT) (black) and Cdkn1c^BAC-LacZ^ (grey—no elevated *Cdkn1c* expression) progressively increased in weight and were not significantly different from one another (n: WT 9, Cdkn1c^BACx1^ 8, Cdkn1c^BAC-LacZ^ 10), ** *p* < 0.01, *** *p* < 0.001; (**b**) the food consumption difference between WT, Cdkn1c^BACx1^ and Cdkn1c^BAC-LacZ^ males on a high-fat diet was not found to be significantly different, (n: 7, 5, 8); (**c**) Glucose tolerance testing of adult male mice completing a high-fat diet challenge. Cdkn1c^BACx1^ (light green) males were found to possess significantly improved glucose clearance compared to WT (black), (n: 9, 8), * *p* < 0.05; (**d**) Insulin tolerance testing of adult male mice completing a high-fat diet challenge. Cdkn1c^BACx1^ (light green) males were found to possess significantly greater sensitivity to insulin challenge between 30–60 min post injection, (n: 9, 8), * *p* < 0.05.

Following the observation that elevated *Cdkn1c* could protect against age-related metabolic syndrome, we challenged Cdkn1c^BACx1^ male mice and litter-matched WT controls with a high fat diet (58V8, TestDiet) from 10 weeks of age for 12 weeks. Cdkn1c^BACx1^ males were found to be highly resistant to weight gain during the dietary challenge (Figure 2a). The average weight gain was 12.4% of the original body weight (2.1 g) over the 12-week period in contrast to the average 41.2% (10.1 g) gained by wild type male controls. In this experiment, we also included a third transgenic line to isolate the contribution of *Cdkn1c* to this resistance against weight gain. This third line of mice called Cdkn1c^BAC-LacZ^ carries one copy of the same BAC transgene but without over-expression of *Cdkn1c*. Any phenotypes observed in Cdkn1c^BACx1^ mice and Cdkn1c^BACx2^ mice that are absent in Cdkn1c^BAC-LacZ^ mice must be due to elevated *Cdkn1c*. Cdkn1c^BAC-LacZ^ male mice gained weight on the high fat diet with the same profile as WT controls (Figure 2b) demonstrating that this protection against weight gain was due to *Cdkn1c* alone. To explore the extent of this phenotype, glucose tolerance testing was performed on these animals, with Cdkn1c^BACx1^ males displaying significantly improved glucose clearance (Figure 2c), compared to wild type mice. Further to this, insulin tolerance testing revealed a heightened response to insulin challenge in Cdkn1c^BACx1^ males (Figure 2d), correlating with the effects observed in aging animals (Figure 1c,d).

**Figure 3:**
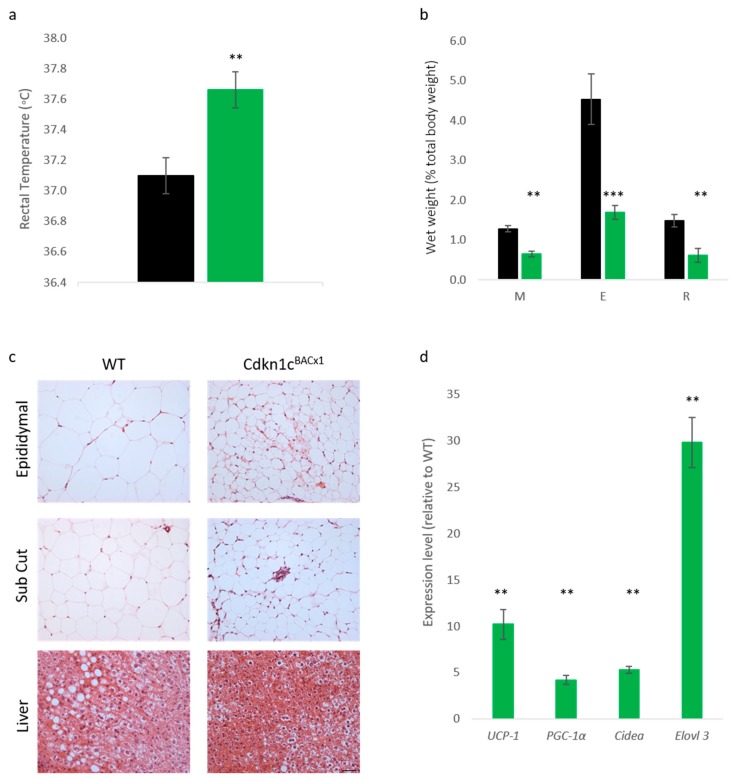
Cdkn1c^BACx1^ mice show reduced lipid stores and elevated brown adipose tissue (BAT) markers in high-fat diet (HFD)-exposed white adipose tissue. (**a**) Rectal temperature of adult male mice exposed to high-fat diet challenge. Cdkn1c^BACx1^ males (light green) had a significantly increased core body temperature when compared to WT littermates (black), (n: 9, 8), ** *p* < 0.01; (**b**) Wet weight of white adipose tissue in high-fat diet exposed males, as a percentage of total body weight. Mesenteric (M), epididymal (E) and retroperitoneal (R) depots were all found to be significantly smaller in Cdkn1c^BACx1^ males (light green) than WT (black). (n: 9, 8), ** *p* < 0.01, *** *p* < 0.001; (**c**) H+E staining of epididymal (top row), anterior sub-cutaneous (Sub Cut, middle row) white adipose and liver (bottom row) in WT (left column) and Cdkn1c^BACx1^ (right column) adult males exposed to high-fat diet challenge. Large unilocular cells were consistently observed in WT adipose depots, with lipid deposits clearly visible in liver sections. Smaller unilocular and multi-locular adipocytes were observed in Cdkn1c^BACx1^ sections, with sparse lipid deposits in liver sections (scale bar: 20 µm); (**d**) Quantitative RT-PCR analysis of brown adipose markers in retroperitoneal white adipose tissue, from adult male mice exposed to a high-fat diet. Significantly elevated markers of brown adipogenesis were detected in Cdkn1c^BACx1^ transcripts when compared to wild type (n: 4, 4). ** *p* < 0.01.

Reduced weight in adult mice possessing excess *Cdkn1c* may, in part,2 be attributed to increased brown adipose tissue content, both in the classic interscapular brown adipose depot but also within white adipose depots, and therefore elevated mitochondrial uncoupling [23]. In order to assess whether the protective phenotype observed in Cdkn1c^BACx1^ mice challenged with a high-fat diet could be attributed to increased BAT content, core temperature was first checked after the 12-week dietary challenge. Cdkn1c^BACx1^ males were found to be significantly hotter than wild type mice (Figure 3a). Upon culling, the wet weights of selected white adipose depots were found to be significantly reduced in the Cdkn1c^BACx1^ mice (Figure 3b), with epididymal (E) and retroperitoneal (R) depots more than 50% smaller than wild type, as a percentage of total body weight. Qualitatively, hematoxylin and eosin (H+E) staining revealed differences in white adipocytes (Figure 3c) with WT epididymal and anterior sub-cutaneous depots containing predominantly larger uni-locular adipocytes relative to the corresponding depots from Cdkn1c^BACx1^ males, which contained areas of multi-locular adipocytes most closely associated with brown adipocytes. In the liver, there appeared to be reduced lipid accumulation (white regions) in Cdkn1c^BACx1^ compared to WT samples. We previously reported the increased expression of a number of BAT markers downstream of the BAT-determining gene *Prdm16* in WAT from young mice, and increased PRDM16 protein but not mRNA, suggesting a function for *Cdkn1c* in the post-transcriptional regulation of *Prdm16* [23]. QRT-PCR analysis of retroperitoneal adipose tissue revealed a higher mRNA expression of *mitochondrial uncoupling protein 1* (*Ucp-1*) and the brown adipose markers *PGC-1α*, *Cidea* and *Elovl3* in Cdkn1c^BACx1^ retroperitoneal depots relative to WT (Figure 3d). While we have not excluded the contribution of other sites of *Cdkn1c* over-expression to the phenotype, taken together, these results are consistent with the increased BAT driven by elevated *Cdkn1c* dosage providing a protective effect against weight gain.

**Figure 4:**
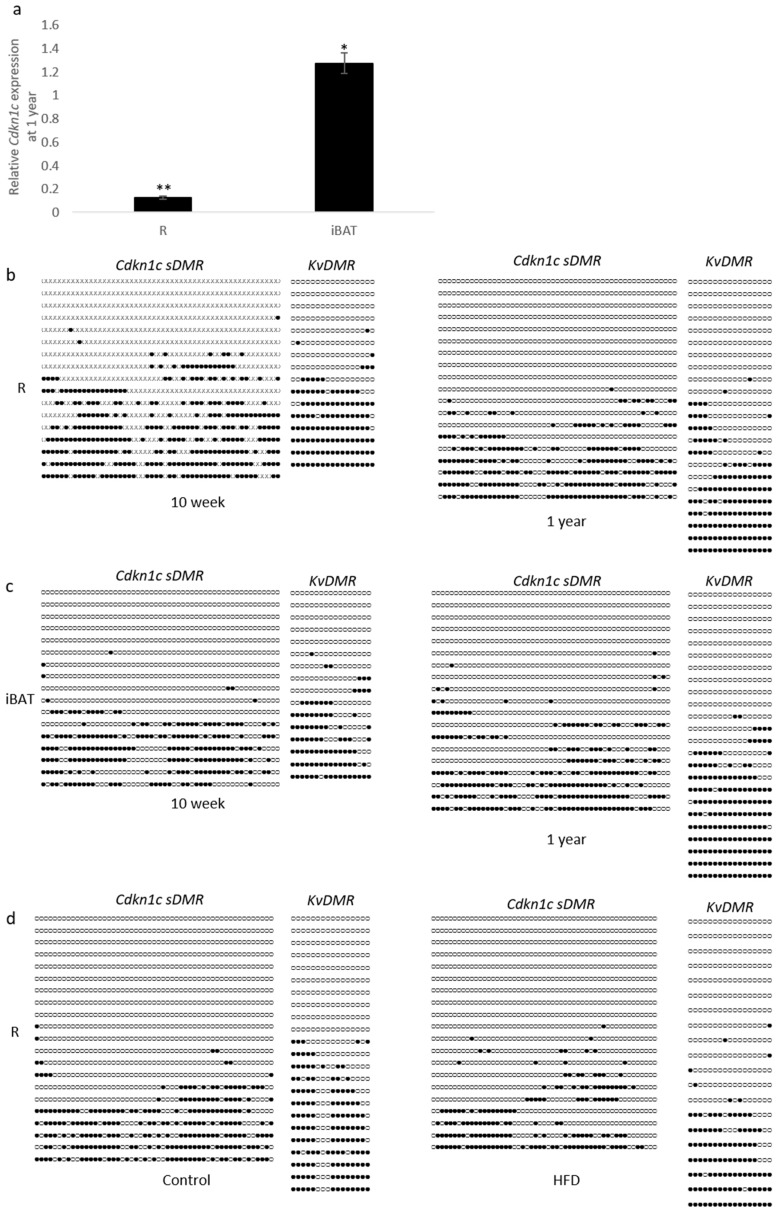
The *Cdkn1c* imprint is stable during aging and during post-natal diet challenge. (**a**) Quantitative RT-PCR analysis of *Cdkn1c* expression changes between 10 weeks and 1 year of age in WT adipose depots. A significantly reduced expression of *Cdkn1c* was detected in aged retroperitoneal (R) adipose tissue, while brown adipose (iBAT) depots were found to possess small but significant increases in *Cdkn1c* expression in aged tissue, (n: 4, 4), * *p* < 0.05, ** *p* < 0.01; (**b**) Representative bisulphite sequencing analysis of *Cdkn1c sDMR* and *KvDMR* 10-week (left column) and 1-year old (right column) retroperitoneal (R) adipose tissue found similar patterns of methylation between timepoints in male mice (n: 2, 2) (Filled circles: Methylated CpG, Unfilled circles: Un-methylated CpG); (**c**) Representative bisulphite sequencing analysis of *Cdkn1c sDMR* and *KvDMR* in 10-week (left column) and 1-year old (right column) iBAT found similar patterns of methylation between timepoints in male mice (n: 2, 2); (**d**) Representative bisulphite sequencing analysis of *Cdkn1c sDMR* and *KvDMR* control (left column) and high-fat diet fed (right column) retroperitoneal adipose tissue (R) methylation, from adult male WT mice. Similar patterns of methylation were found between diets (n: 2, 2).

Recent work has shown that the epigenetic marks which silence the paternal allele of *Cdkn1c* can be disrupted through protein restriction during gestation [17]. Further to this, the stability of imprints during aging has recently come into focus, with several reports of altered DNA methylation in aged samples [26,27]. We examined *Cdkn1c* expression in retroperitoneal white adipose tissue (R) and interscapular brown adipose tissue (iBAT) in 10-week old WT mice compared to 12-month old mice from the same colony. Older mice showed significantly reduced expression of *Cdkn1c* in white adipose and slightly elevated expression in brown adipose tissue compared to younger mice (Figure 4a). Two regions of differential methylation (*Cdkn1c sDMR* and *KvDMR*) that regulate *Cdkn1c* expression were examined through bisulphite sequencing of genomic DNA from the same individuals to try and account for these observed changes in transcription. Methylation of the *Cdkn1c sDMR* and *KvDMR* in early life adipose tissue has previously been shown to be similar to that seen in other tissue [23]. A qualitative analysis of 10-week (left column) and 1-year old (right column) wild type retroperitoneal adipose tissue revealed a similar pattern of methylation at both *Cdkn1c sDMR* and the *KvDMR* (Figure 4b). No gross differences were observed in brown adipose tissue (Figure 4c), where expression had been found to be modestly elevated in 1-year old samples (Figure 4a). Finally, samples were analyzed from the high-fat diet cohort, with no gross differences in methylation detected after 12 weeks of dietary challenge in retroperitoneal tissue (Figure 4d).

## 3. Discussion

In this study, we show that mice with a single extra copy of *Cdkn1c* are able to resist both age and diet-driven weight gain and maintain a healthier management of glucose than matched controls. While we have not defined the mechanism underlying this phenotype, the gains in brown adipose tissue that we have shown in younger mice [23], alongside the higher body temperature and increased expression of markers of brown fat, suggest that increased thermogenesis may, at least in part, play a contributory role.

Using both loss-of-expression and gain-in-expression animal models, alongside ex-vivo approaches, we previously identified an important and intrinsic role for *Cdkn1c* in the development of both classic brown adipose tissue and the brown-like adipose tissue that develops post-natally within white adipose depots [23]. We showed that mice with a 2–3-fold elevated expression of *Cdkn1c* possessed substantially more brown adipose tissue and substantially less white adipose tissues at 10 weeks of age. In this new study, we show that mice from these same transgenic lines do not gain significant weight as they age, unlike their wild type littermates, and this was true for both males and females. We also show that these older mice are better able to manage their glucose levels. A caveat to this observation is the high blood glucose values relative to other studies on this strain background [28]. This may be explained by the standard chow on which we routinely maintain mice, Labdiet 5008, which provides a relatively higher proportion of energy from fat and carbohydrate compared to other standard chow diets and may induce a diabetic state when fed over a prolonged period. An alternative and potentially more interesting explanation is that the WT mice were inherently stressed, a factor known to impact blood glucose levels [29]. Higher stress levels in the WT littermate controls is suggested by our previous observation that male Cdkn1c^BACx1^ mice disrupt social stability when co-housed with WT mice, resulting in an increased incidence of fighting and wounding within these groups [30]. Further studies including single housing animals will be required to fully explore these differences.

We further show that younger mice exposed to a high-fat diet for 12 weeks gain almost no weight despite consuming a similar amount of HFD as their WT counterparts under the same protocol. As with the older mice, the high-fat diet-exposed *Cdkn1c* transgenic mice were also better able to manage their glucose levels than the controls. Using a control line of mice (Cdkn1c^BAC-LacZ^) which carried the same BAC transgene but without over-expression of *Cdkn1c*, we were able to attribute this weight resistance phenotype to elevated *Cdkn1c*. Taken together, these data highlight a previously unappreciated role for *Cdkn1c* in weight regulation later in life, at least in mice. The loss or gain in function of *CDKN1C* in humans is already implicated in the childhood growth disorders Beckwith-Wiedemann Syndrome, IMAGe syndrome and SRS, recapitulated to some extent in animal models. This new data highlights a role for *Cdkn1c* in regulating adiposity in the adult mouse. Quite remarkably, only a two-fold elevation in expression of *Cdkn1c* was able to protect mice against both age and diet-induced weight gain. It should be noted that the changes we observed were between 6 months and 1 year of age, which is thought to represent middle age in mice. Our analyses did not extend to cover the full aging period. It will be interesting to follow these mice for an extended period into old age to ask whether they maintain this metabolic advantage, and whether they have an extended lifespan. Further to this, while a reduction in body weight was observed in both genders, a dosage dependent phenotype was not so clearly observed in females. Variation in the individual weights of the transgenic groups was larger at 12 months than those observed in male mice (Figure 1a, b). It will be important to explore this potential difference between genders in a larger cohort over a longer time period.

The inflexibility of the *Cdkn1c* imprint to either aging or post-natal diet modification contrasts with data seen from *in utero* exposure [17]. We found that erosion of the *Cdkn1c sDMR* occurred in offspring exposed to gestational low protein diet, with these changes maintained post-natally. Our current data would therefore lend support to the hypothesis that altering the dosage of *Cdkn1c* in response to gestational environmental factors can be achieved, but once laid down, this methylation is resistant to post-natal adjustment. However, we analyzed DNA methylation in a small number of individuals, so we cannot exclude more modest changes in DNA methylation with time or changes in other tissues or other strains of mice not analyzed. This stubbornness to change likely provides a protective effect in mice, as has been highlighted by the differences in weight observed in our loss of imprinting model (Cdkn1c^BACx1^). A single extra copy of *Cdkn1c* was sufficient to restrict mice in both aging and diet models to only modest weight gain, in addition to the embryonic and post-natal growth restriction previously described [20]. In a laboratory setting, such responses would appear advantageous. However, outside of food available *ad libitum*, such excessive uncoupling may in fact reduce survival chances, due to the challenges in laying down sufficient adipose stores. By restricting changes in imprinted status to an embryonic window, rather than a constant environmental sensitivity, this might be prevented.

*Cdkn1c* has only recently been identified as having a role in adipose tissue development [23]. Interestingly, the response to glucose and insulin tolerance testing in aging mice was found to be largely comparable between the Cdkn1c^BACx1^ and Cdkn1c^BACx2^ line, whereas whole body and adipose depots weights were found to be dosage dependent, with the Cdkn1c^BACx2^ line having even greater reductions. This dosage-based phenotype is also present at early post-natal and fetal time points [20]. The lack of a dosage-based phenotype for glucose and insulin tolerance may be attributed to simply avoiding the age-related metabolic syndrome that is observed in wild type mice, due to their leanness, rather than substantial changes to insulin signaling. However, epigenetic dysregulation at the IC2 cluster has previously been demonstrate to affect β-cell mass [31], and therefore a direct role cannot be excluded.

In summary, this is the first report linking *Cdkn1c* dosage to weight gain in adult mice. Our findings, if they translate to humans, have potentially monumental implications for human health. In addition to the potential relevance to the rare human disorders that involve either elevated expression of *Cdkn1c* or gain in function mutations of this gene, SRS and IMAGe syndrome [18], worldwide obesity has nearly tripled in the last 40 years, resulting in over 650 million obese adults and an estimated 41 million children under the age of 5 years being overweight or obese (WHO). Unlike many other genes implicated in weight regulation, *Cdkn1c* is epigenetically regulated. This suggests the possibility that epidrugs could be developed that activate the silent *Cdkn1c* allele with therapeutic benefit. *Cdkn1c* encodes a cyclin-dependent kinase inhibitor and, while we have not identified the molecular mechanism through which *Cdkn1c* drives the development of brown adipose, it may be possible to develop designer drugs to mimic this activity. Ultimately, drugs aimed at *Cdkn1c* may provide a way to increase BAT thermogenesis in adult humans to increase whole-body energy expenditure and address the global epidemic of obesity.

## 4. Materials and Methods

### 4.1. Animals and Husbandry

All animal studies and breeding were approved by the University of Cardiff ethical committee and performed under a UK Home Office project license (30/2600 from 4 November 2007 until 4 November 2013). Mice were housed on a 12 h light–dark cycle with lights coming on at 06.00 h with a temperature range of 21 ± 2 °C with free access to tap water and standard chow (Labdiet 5008, Labdiet, St. Louis, MO, USA). BAC transgenic lines Cdkn1c^BACx1^ (previously known as 5D3; single copy of unmodified BAC), Cdkn1c^BACx2^ (previously known as 5A4; two copies of unmodified BAC) and Cdkn1c^BAC-LacZ^ (previously known as 10–10; single copy of modified BAC with no transgenic elevation of *Cdkn1c*) were bred onto a C57BL/6J (BL6) background for >12 generations and genotyped as described [21]. For aging studies, mice were group housed and weighed monthly. For feeding studies, grouped male mice were transferred onto 58V8 diet (TestDiet, St. Louis, MO, USA) for 12 weeks, with weights measured weekly. Core body temperatures of high fat diet mice were monitored with a rectal probe (IN005A, Vet-Tech solution, Congleton, UK).

### 4.2. Glucose Tolerance Testing

Mice were fasted overnight, with access to water. All subjects were individually housed for the duration of the test, and were weighed before testing began. Tail blood was taken and glucose concentration was recorded by Glucose 201+ meter (HemoCue, Ängelholm, Sweden). After samples were taken, mice were injected intraperitoneally with 10 mM/ kg (1.8 mg/g) glucose (Sigma Aldrich, St. Louis, MO, USA) and further readings were taken at 30, 60, 90 and 120 min post-injection. All experiments were performed blind.

### 4.3. Insulin Tolerance Testing

Mice were individually housed with access to food and water. Tail blood was collected and glucose concentration was recorded by Glucose 201+ meter (HemoCue, Ängelholm, Sweden). After samples were taken, mice were injected intraperitoneally with 0.75 U/ kg insulin (Novo Nordisk Bagsvaerd, Denmark) and further readings were taken at 15, 30, 45 and 60 min post-injection. All experiments were performed blind.

### 4.4. DNA, RNA and H+E Staining Analysis

Genomic DNA was bisulphite treated using an EZ DNA Methylation Kit (Zymo Research, Irvine, CA, USA). Sodium modification treatments were carried out in duplicate for each DNA sample and at least three independent amplification experiments were performed for each individual examined. The region spanning the *Cdkn1c sDMR* was amplified by PCR using primers 5′-tgggtgtagagggtggatttagtta-3′ and 5′-cccacaaaaaccctaccccc-3′ and hemi-nested primer 5′-gtattgttaggattaggatttagttggtagtagtag-3’. For amplification of KvDMR, the following primers were used 5′-taaggtgagtggttttaggat-3′ and 5′-aatcccccacacctaaattc-3′ and hemi-nested primer 5′-ccactataaacccacacata-3′. TaKaRa EpiTaq™ HS (Takara, Shiga, Japan) was used for PCR amplification according to the manufacturer’s specifications. The PCR products were cloned into pGEM-T (Promega, Madison, WI, USA) and an average of 20 clones per sample were sequenced using M13 reverse primer and an automated ABI Prism 3130xl Genetic Analyzer (Applied Biosystems, Foster City, CA, USA) as previously described [16]. Quantitative RT-PCR was performed in duplicate on four independent samples as previously described [32]. Briefly, total RNA was prepared from tissues using RNA-Bee (AmsBio, Abingdon, UK). cDNA was prepared using M-MuLV reverse transcriptase (Roche, Basel, Switzerland) with random hexamers under the manufacturers conditions. Quantitative RT-PCR was performed in duplicate on four independent samples using DyNAmo HS SYBR Green qPCR kit (Finnzymes, New York, NY, USA) against the reference gene *β-Actin*, and detected using the Chromo 4 Four-Color Real Time detector (MJ Research, Reno, NV, USA). H+E staining was performed as previously described [21]. At least three samples per experimental group were fixed overnight in phosphate-buffered 4% paraformaldehyde, paraffin-embedded and 7 μm sections cut, prior to H&E staining. Images are representative of each experimental group.

### 4.5. Primers for qRT-PCR

*Cidea F: 5′*-tggaaaagggacagaaatgg *R: 5′*-tctcgtacatcgtggctttg
*PGC-1α F: 5′*-tcatcacctaccgttacacctg *R: 5′*-caagcttctctgagcttccttc
*Elovl3 F: 5′*-ctgttgctcatcgttgttgg *R: 5′*-atctgactacggcgtcatcc
*Cdkn1c F: 5′*-agagaactgcgcaggagaac *R: 5′*-tctggccgttagcctctaaa
*Ucp-1 F: 5′*-ggcaaaaacagaaggattgc *R: 5′*-taagccggctgagatcttgt
*β-Actin F: 5′*-cctgtatgcctctggtcgta R: *5′*-ccatctcctgctcgaagtct

### 4.6. Statistical Analyses

Statistical significance (probability values) was determined using the Student’s *t*-test (two-tailed distribution and two sample unequal variance). For quantitative RT-PCR analysis, the Mann-Whitney test was performed on Δ*C*_t_ values between groups.
